# Deeper Sections: Its Frequency and Diagnostic Utility in Histopathology of Noncutaneous Small Biopsy Specimen in a Tertiary Hospital in Nepal

**DOI:** 10.1155/2021/5926047

**Published:** 2021-08-28

**Authors:** Gopal Lama, Paricha Upadhyaya, Smriti Karki, Anju Pradhan

**Affiliations:** ^1^Department of Pathology, B.P. Koirala Institute of Health Sciences, Dharan, Nepal; ^2^Department of Pathology, Rapti Academy of Health Sciences, Ghorahi, Nepal

## Abstract

**Background:**

Biopsy is an important tool for accurate diagnosis of disease in histopathology which can be examined at multiple levels during microscopic examination. The initial sections may not be representative of the entire biopsy, which leads to frequent request for deeper sections. This study assessed the frequency and diagnostic utility of deeper sections in noncutaneous small biopsy specimens at a tertiary hospital. *Material and Methods*. All the formalin fixed noncutaneous small biopsy specimens received were processed for grossing followed by tissue processing and embedding. The paraffin-embedded blocks were cut into 3–5 *µ*m sections, fixed in a glass slide, deparaffinised, and then stained with hematoxylin and eosin (H&E) stain as per the standard protocol. Deeper sections were instructed based on microscopic findings of initial slides. The overall frequency of deeper section, its levels of cutting, tissue survival, and outcome were assessed and interpreted for the final diagnosis.

**Results:**

A total of 125 cases (26.9%) from 464 samples received were requested for deeper sections. The most frequent deeper sections were from cervix (72 cases) followed by stomach (18 cases) and endometrium (17 cases). The deeper sections were performed most frequently at 4 levels (17.8%) followed by 5 levels (14.8%) and 6 levels (13.3%). Deeper sections revealed nondiagnostic additional features in 28.2%, while 2.2% showed additional diagnostic features. Likewise, 2.2% had tissue segment loss. The overall mean level showing additional features was 6 levels. Tissue survival increased in 13% cases and 1.5% had decreased survival. The most common reason for requesting deeper section was to enhance sensitivity and diagnostic accuracy of disease.

**Conclusions:**

Deeper sections often contribute to final diagnosis. Therefore, deeper sections on biopsies that cannot readily be diagnosed on routine levels are recommended regardless of size of the lesion and there should be uniformity in the practice of deeper sections across the globe.

## 1. Introduction

A biopsy is a medical test in which sample tissues are extracted from a living body for examination under microscope and to determine the presence or extent of the disease by a pathologist [1, 2]. Proper histopathological examination of biopsied tissue is the gold standard for the diagnosis of many lesions including malignant, inflammatory, and infectious diseases [3] and thus can be used for diagnostic, prognostic, and treatment planning purposes [1, 2].

Small biopsy samples are those tissues, few mm to cm in size, that rarely need dissection and can be simply processed, embedded, and sectioned as they are present [4, 5]. A majority of biopsy tissues can be adequately examined at multiple levels allowing the pathologist to assess the tissue as a whole during microscopic examination [4]. Deeper sections are frequently requested to enhance the sensitivity and diagnostic accuracy of the diseases [6]. Varieties of sections can be opted based on the need such as step section, multiple step section, deep cut, or better section [7–9].

Determination of the optimum number of sections and/or levels in small biopsies has been the subject of several studies. Many have recommended 3 to 4 levels for optimum sectioning [10–12] but it has been difficult to draw generalized conclusions. In colorectal polyp, a depth of at least 3–6 levels is advised [13, 14]. However, in some other studies, deeper sections of initially negative colorectal biopsy specimens have revealed tubular adenomas even at levels 7 and 8 [15]. Another study on nondiagnostic colorectal polyp biopsy showed that 10% of cases had diagnostic findings when deeper sections were performed up to 380 *μ*m [16].

Like in many hospitals, deeper sections are regularly practiced in our laboratory setup as well. Deeper sections have been found to be helpful in providing additional information during the final diagnosis of small biopsy specimens, thereby increasing the diagnostic accuracy leading to subsequent improvement in overall patient care [8, 9, 14–16]. This study has therefore been conducted to find the overall frequencies and levels of deeper section requested for small biopsy specimens of various organs and the factors warranting its need. Similarly, this study also assesses the survival of tissues as well as the final outcome in deeper sections.

## 2. Material and Methods

### 2.1. Design and Study Samples

This is a cross-sectional study conducted at the Department of Pathology, BP Koirala Institute of Health Sciences, Dharan, Nepal, over a period of one year (September 2017–September 2018). The study included the noncutaneous small biopsy specimens from the gastrointestinal tract (GIT) and female reproductive system, received at the Department of Pathology. Specimens from other organ system were not included in this study due to limited sample size. Formalin (10%) fixed small biopsy specimens received were processed for grossing followed by tissue processing and embedding. The paraffin-embedded blocks were cut into 3–5 *µ*m sections, fixed in a glass slide, and deparaffinised and then hematoxylin and eosin (H&E) staining was done. The slides were viewed under light microscope by the consultant histopathologist for microscopic findings. Deeper sections were instructed as indicated based on microscopic findings of initial slides and clinical history and were reviewed for the interpretation of final diagnosis. We in our study independently reviewed all the slides to check for additional information contributed by the deeper sections.

In order to avoid bias, various operational definitions were considered as different types of deeper sections practiced in this institution and around the world. Sections >5 *µ*m taken to analyse deeper tissues were considered deeper sections that included the following (a) step section or step cut (SC)—section taken after each alternate section; (b) 2-step section (2SC)—section taken after every two sections; (c) 3-step section (3SC)—section taken after every third sections; (d) multiple step/serial sections (MSS)—sections taken after at least three regular sections; (e) deep cut (DC)—any deeper section taken depending upon the reserve tissue present; (e) better section (BS)—section taken (generally one) using new blades; (f) thin section (TS)—section taken <3 *µ*m in thickness; and (g) 1 level defined as 1 section in the slide.

## 3. Observation and Results

### 3.1. Frequency of Deeper Sections

A total of 464 noncutaneous small biopsy specimens from the two organ systems (female reproductive (407) and gastrointestinal tract (57)) were included in the study. Deeper sections were performed in 125 (26.9%) cases of which 89 cases were from the female reproductive system (72 cases from cervix and 17 cases from endometrium). Similarly, 36 cases from gastrointestinal tract (GIT) had deeper sections of which 6 cases were from oesophagus, 18 cases from stomach, 4 cases from duodenum, and 8 cases from colorectal.

Different types of deeper sections used separately or in combination were performed. The most frequently requested type of deeper section was DC (36.3%) followed by BS (21.5%), MSS (20.7%), 3SC (15.6%), SC (8.1%), 2SC (6.7%), and TS (2.2%).

### 3.2. Assessing Diagnostic Utility of Deeper Sections in Final Diagnosis

**Figure 1:**
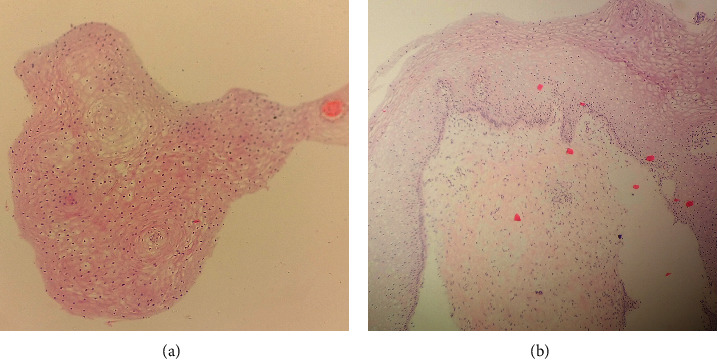
(a) Cervical biopsy. Initial section showing strips of ectocervical lining epithelium only (H&E stain (100X)). (b) Cervical biopsy. Deeper section (level 7) showing ectocervical tissue with features of chronic cervicitis (H&E stain (100X)).

Outcomes of deeper sections were assessed. 67.4% had similar findings to those of initial section. There was more clear morphology or additional tissue features supporting the final diagnosis in 28.2% but without altering the initial diagnosis. Similarly, 2.2% had tissue segment loss compared to the initial sections. Another 2.2% revealed additional features in deeper section which altered the initial diagnosis: from reactive atypia to high grade squamous intraepithelial lesion (HSIL) in one case and from initial superficial nondiagnostic findings (Figure 1(a)) to chronic cervicitis (Figure 1(b)) in two cases.

### 3.3. Reasons for Deeper Sections

It was found that 93.3% of deeper sections were instructed to enhance the sensitivity and diagnostic accuracy. These were done for disclosing additional pathological findings and hidden malignancies in 63.7%. Similarly 34.8% deeper sections were done because initial sections were too superficial and nondiagnostic. In 23.7%, it was done for having unusual findings seen in initial sections from that of provisional clinical diagnosis. For instance, a clinician may send cervical biopsy tissue which was diagnosed as HSIL in Pap smear, but histology examination in initial section may show normal findings or sometimes may show tissues from different organ like endometrial tissue instead of cervical tissue.

In 5.2%, deeper sections were requested to rule out the artefacts. Likewise, 2.2% were done for each orientation and plane of sectioning the tissue specimen as well as to know the clearance of surgical margins and the extent of invasion, respectively.

### 3.4. Deeper Section Levels and Frequency per Organ System

Deeper section levels of cervix showed that 18.1% had 5 levels, 13.9% had 6 levels, 12.5% had 4 levels, 11.1% had 3 levels, 9.7% had 7 levels, 8.3% had 8 levels, 5.6% had sections taken at 9 levels and 11 levels each, 4.2% had sections at 10 levels and 12 levels each, and 2.8% had 13 levels while 1.4% had sections taken at 2 levels, 19 levels, and 20 levels each.

Deeper section levels of endometrium were done in 41.2% at 4 levels and 11.8% at 1 level, 2 levels, 3 levels and 6 levels each while 5.9% had deeper sections at 5 levels and 12 levels each, respectively.

Deeper section levels from upper GIT were performed in 21.4% at 6 levels, 18% had 5 levels, 11% had 8 levels, and 7.1% had deeper sections at 3 levels, 7 levels, 9 levels, 10 levels, and 13 levels each, while 3.6% had sections taken at 11 levels, 14 levels, 15 levels, and 22 levels each. Deeper section levels of lower GIT showed that 75% had 6 levels while 12.5% had 3 levels and 7 levels each.

### 3.5. Assessing Diagnostic Utility of Deeper Sections in Final Diagnosis per Organ System

Deeper section levels showing additional features per organ system were analysed (Table 1). Twenty-five cases from cervix showed additional features in deeper sections of which ten cases (five cases each) had 4 levels and 3 levels, six cases (three cases each) had 7 levels and 5 levels, and six cases (two cases each) had 11 levels, 8 levels, and 6 levels while three cases (one case each) had 10 levels, 9 levels, and 2 levels, respectively, showing additional features. Eight cases from stomach showed additional features in deeper section; three cases at 6 levels while one case each at 22 levels, 10 levels, 8 levels, 7 levels, and 5 levels, respectively. Four cases from endometrium showed additional features in deeper section, 2 cases at 2 levels and one case each at 6 level and 4 levels. Three cases from colorectal biopsies showed additional features in deeper sections at 4 levels each.

Mean of deeper section levels per organ system revealing additional features was also analysed (Table 2). The overall mean of deeper section was 5.5 levels.

### 3.6. Assessment of Tissue Survival

**Figure 2:**
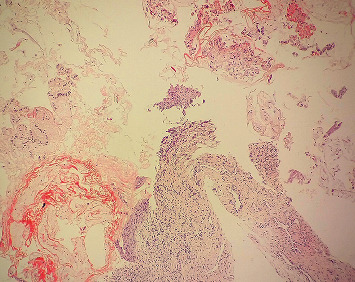
Tissue fragmentation. Microscopic examination shows multiple bits of tissue from cervix (at grossing: 2 bits of tissue) (H&E stain (100X)).

Assessment of tissue survival in microscopic examination revealed that 85.9% had similar findings while 12.6% had increased tissue retrieval (Figure 2). However, 1.5% had decreased number of tissues compared to those found in gross examination.

## 4. Discussion

In our study, different types of deeper sections with 3–5 *µ*m thickness were taken at a fixed distance after reviewing the initial slides as per the judgement of the reporting pathologist. These deeper sections were used as better section, deep cut, step sections, or multiple serial sections, and each section was subsequently labelled as levels. For instance, one step section was labelled as 1 level, whereas 3 step sections were labelled as 3 levels. Each level was thoroughly examined for any additional feature to those of initial sections. Significant findings which improved the diagnosis were seen at varied levels in 28.2% of the cases, while 2.2% of the cases had additional diagnostic findings. Thus, deeper sections in small biopsies are commonly practiced to enhance the diagnostic sensitivity and accuracy as is evident in our results (40 cases had additional findings in deeper sections) as well as in other studies [7, 14].

### 4.1. Frequency of Deeper Sections

Our study showed that a total of 125 cases (26.9% of the total 464 cases) had deeper sections at various levels, compared to 10% in Patil et al.'s [8] study and 8% in Manyam et al.'s [7] study. This shows that our study had a slightly higher frequency of deeper sections which can be assumed owing to the fact that our study included noncutaneous biopsy samples from two different organ systems unlike in the other two studies which used only the similar cases from oral histopathology archive. This value of 26.9% is only a proportionate representation of the biopsy load received in our pathology laboratory.

### 4.2. Types of Deeper Section

Different types of deeper sections were used in our study, in combination or as single entity, as per the reporting consultant pathologist during the routine histopathological examination. The request of different types of deeper sections or the levels of deeper sections was solely dependent upon the reporting pathologist's judgement as there was not any common consensus regarding this among the pathologists in our institution or other laboratories across the globe. We observed a very nonjudicious use of terminology of deep sections wherein it could have a completely different meaning when we take into account different types of biopsy specimens. Literature review revealed that most of the other studies have used a single deeper section type at a uniform depth and number of levels. Like, step serial sections have been used in studies by Schick et al. [17], Nichols et al. [18], and Chitkara et al. [12]. However, discrepancies in depth of levels were seen in these studies as well as other studies even though they used the same terminologies. Step serial section means each section taken at 28 *µ*m to 64 *µ*m apart in Chitkara et al.'s [12] study, while sections are 50 *µ*m apart in Schick et al.'s [17] study, but step serial means each section taken 5 *µ*m apart for us which we have been practicing for years in our laboratory.

Similarly, a study by Mahjoub et al. [19] used deep section (DC) only. Luo et al.'s [10] and Parameswaran et al.'s [14] studies used step sections (SC) each at 1/3^rd^, 40%, and 60% into the block, while Nielsen et al. [13] used step sections each taken at 25%, 50%, and 75% of the tissue block. Here, also the discrepancy in terminology and its interpretation is evident. Likewise, Wu et al. [16] used step sections as well as multiple serial sections in combination. A study by Geisinger et al. [20] that surveyed on gastrointestinal pathologist regarding deeper sections usage on esophageal biopsy showed that most pathologists preferred 3 SC followed by multiple serial sections when requesting deeper sections. Multiple serial sections will allow the pathologist to get more detailed information in each level than step sections in which we discard the sections at certain interval. Due to this, some diagnostic lesion could be missed; on the contrary, step sections can allow the remaining sections to be used for other purposes like for special stains [8]. Similarly, better section will minimize the artefacts like shattering effects caused by blunt blades. At our institute, it is practiced by changing a new microtome knife to get a better section without shattering effect, whereas we could not find the use of better section in any of the literature reviews we conducted during this study. In this era of global transmigration of human resource, it is vital that we understand the meaning of a terminology equally. Therefore, there is a need to establish a protocol wherein we communicate clearly with the technical staff in terms of level of cuts that we would desire.

### 4.3. Level of Deeper Sections

Different studies have used varied levels of deeper sections. In our study, the range of levels included from level 1 to level 22 with the mean at level 6. The deepest we went was for a case of gastric biopsy and we definitely had a significant change, because the initial section did not have any mucosal tissue and finally at 22^nd^ level we got a glimpse of foveolar epithelial lining. However, this additional finding did not add or alter the diagnostic value to the slide since the requisition was made in order to look for an ulcer. Other studies have gone up to 380 microns [16], which is much deeper than those in our study.

Though the depth of levels was partly dependent on the size of the biopsy specimen, it was still as multiples of five (5 *µ*m), so we still had some kind of uniformity in deeper section levels unlike many other studies. In the study conducted by Chitkara et al. [12], we have observed that the gap between levels was not uniform ranging from 28 *µ*m to 64 *µ*m apart. Likewise, Schick et al. [17] used 3 to 10 levels each at 50 *µ*m apart and Nielsen et al. [13] used 3 levels, each taken from 25%, 50%, and 75% into the block. Similar other studies conducted by Wu et al. [16], Warnecke et al. [21], Nagata et al. [9], and Nash et al. [15] have also used varied levels.

### 4.4. Utility of Deeper Sections

**Figure 3:**
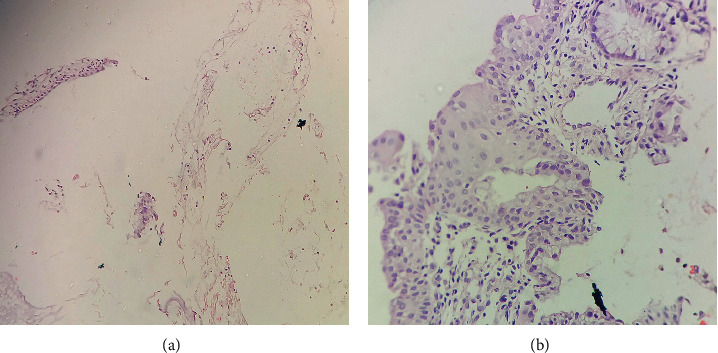
(a) Cervical biopsy. Initial section showing predominantly mucous flakes (H&E stain (100X)). (b) Cervical biopsy. Deeper section showing features of chronic cervicitis with squamous metaplasia (H&E stain (400X)).

Our study showed that 67.4% of the cases had similar findings in deeper sections. However, 2.2% revealed distinct additional diagnostic features in the deeper sections. One case which had been diagnosed as reactive atypia of cervix was diagnosed as HSIL after reviewing the deeper sections up to 11 levels. Likewise in two cases where initial sections showed only mucus flakes (Figure 3(a)) and haemorrhage, deeper section revealed cervical tissues with features of chronic cervicitis each at 2 levels (Figure 3(b)) and 7 levels, respectively. This was in coherence with Parameswaran et al.'s [14] study (1.7%). In a study done by Luo et al. [10] in cervical biopsies, it was seen that 17% of the cases had discrepancy in findings with initial sections when deeper sections were taken at 40% to 60% into the block which corresponds to approximately >100 levels as of our study. However, we never reached that level in any of the biopsies. Partly this was because we did not deviate from our routine practice and we were not ready to try out deeper levels since this added to the turnover time as ours was a cross-sectional study.

In 2.2% of the cases, initial sections were as good or even better as there was tissue segment loss in deeper sections which showed coherence to Parameswaran et al.'s [14] study (1.3%) and Yadav et al.'s [6] study (3.6%). This shows that deeper sections are not always a boon. Therefore, care must be taken on getting the deeper sections and close level cutting would probably solve this issue of tissue segment loss in deeper sections rather than leaping between the microns.

### 4.5. Tissue Retrieval

Our study also observed that deeper sections in 12.6% of the cases had increased number of tissues in microscopic examination compared to gross examination. This might be due in part to sample fragmentation [17]. Likewise, 1.5% of the cases had decreased tissue survival in microscopic examination which could have been due to improper tissue handling during either grossing, processing, or embedding. Thus, proper handling of biopsy samples during grossing and embedding can minimize the change in tissue survival in microscopic examination, preventing valuable information loss.

## 5. Conclusion and Way Forward

1. The present study shows that deeper sections are helpful in providing additional information and can have a significant impact in the final diagnosis, prognosis, and treatment planning purposes. This is especially true in low income countries where recent advances in diagnostic testing such as immunohistochemistry and molecular genetics are still out of reach in majority of pathology laboratories, and diagnosis is solely based on histomorphology of the tissues examined under microscope. However, different organ systems may require different levels of depth which is often dependent on the amount of tissue left in the block.
2. At today's global era of transmigration of health personnel including pathologists, a common terminology for deeper sections is mandatory. This study has tried to pave the way for standardizing the nomenclature which could be disseminated to the pathologic scientific community for uniformity in practice of deeper section and encouragement to further similar research.

## Figures and Tables

**Table 1:** Frequency of deeper section levels showing additional features per organ system.

SN	Organ system	Level											
		2	3	4	5	6	7	8	9	10	11	22	Total
1	Cervix	1	5	5	3	2	3	2	1	1	2	0	25
2	Stomach	0	0	0	1	3	1	1	0	1	0	1	8
3	Endometrium	2	0	1	0	1	0	0	0	0	0	0	4
4	Colorectal	0	0	3	0	0	0	0	0	0	0	0	3
Total		3	5	9	4	6	4	3	1	2	2	1	40

**Table 2:** Mean levels of deeper sections showing additional features.

Organ system	Cases	Mean	Median
Cervix	25	5.7	5.0
Stomach	8	8.8	6.5
Endometrium	4	3.5	3.0
Colorectal	3	4.0	4.0
Total	40	5.5	4.6

## Data Availability

Any relevant data used to support the findings of this study can be made available upon requests for data, 6 months after publication of this article, and will be considered by the corresponding author.

## Additional Points

The data were entered in Microsoft Excel 2013, and using descriptive statistical analysis, frequencies, percentages, mean, and median were analysed.
